# Predicting conversion to Alzheimer's disease among individual high‐risk patients using the Characterizing AD Risk Events index model

**DOI:** 10.1111/cns.13371

**Published:** 2020-04-03

**Authors:** Xiang Lu, Jiu Chen, Hao Shu, Zan Wang, Yong‐mei Shi, Yong‐gui Yuan, Chun‐ming Xie, Wen‐xiang Liao, Fan Su, Ya‐chen Shi, Zhi‐jun Zhang

**Affiliations:** ^1^ Department of Neurology School of Medicine Affiliated ZhongDa Hospital Southeast University Nanjing China; ^2^ Institute of Neuropsychiatry The Affiliated Brain Hospital of Nanjing Medical University Nanjing China; ^3^ Department of Psychosomatics and Psychiatry Affiliated ZhongDa Hospital of Southeast University Nanjing China; ^4^ Department of Psychology Xinxiang Medical University Xinxiang China

**Keywords:** Alzheimer's disease, biomarker, late‐onset depression, mild cognitive impairment, progression

## Abstract

**Aims:**

Both amnestic mild cognitive impairment (aMCI) and remitted late‐onset depression (rLOD) confer a high risk of developing Alzheimer's disease (AD). This study aims to determine whether the Characterizing AD Risk Events (CARE) index model can effectively predict conversion in individuals at high risk for AD development either in an independent aMCI population or in an rLOD population.

**Methods:**

The CARE index model was constructed based on the event‐based probabilistic framework fusion of AD biomarkers to differentiate individuals progressing to AD from cognitively stable individuals in the aMCI population (27 stable subjects, 6 progressive subjects) and rLOD population (29 stable subjects, 10 progressive subjects) during the follow‐up period.

**Results:**

AD diagnoses were predicted in the aMCI population with a balanced accuracy of 80.6%, a sensitivity of 83.3%, and a specificity of 77.8%. They were also predicted in the rLOD population with a balanced accuracy of 74.5%, a sensitivity of 80.0%, and a specificity of 69.0%. In addition, the CARE index scores were observed to be negatively correlated with the composite Z scores for episodic memory (*R* ^2^ = .17, *P* < .001) at baseline in the combined high‐risk population (N = 72).

**Conclusions:**

The CARE index model can be used for the prediction of conversion to AD in both aMCI and rLOD populations effectively. Additionally, it can be used to monitor the disease severity of patients.

## 1 INTRODUCTION

Early pathological changes in Alzheimer's disease (AD) predate clinical diagnosis by many years.^1^ Therefore, early recognition of individual high‐risk patients likely to develop AD is critical for targeted clinical interventions.^2^ Amnestic mild cognitive impairment (aMCI) is considered the most common disease subtype for progressing of AD.^3^ Studies have shown that aMCI converts to AD from 10% to 15% per year, depending on the country and population studied.^4^, ^5^ Late‐onset depression (LOD), one type of late‐life depression (LLD), represents a major depressive episode that initially occurs after the age of 50‐65.^6^ Importantly, the LOD is suggested to exhibit higher sensitivity to expedited brain aging and likely to predispose to AD through the exhaustion of the brain's structural and functional reserve.^7^ Individuals with LOD commonly display cognitive deficits, and LOD confers a nearly 50% upregulated risk for dementia growth.^8^, ^9^ Furthermore, cognitive dysfunction is reported to be persistent in LOD cases even when remission had been achieved.^10^ In certain aspects, aMCI and LOD confer a high risk of developing AD and they might represent a potential clinical continuum.^11^ Many studies have supported the notion that LOD and aMCI share common AD pathology biomarkers (eg, amyloid‐β[Aβ]),^12^ common genetic risk factors (eg, *APOE* ε4, clusterin‐T allele),^13^, ^14^ common regions of brain atrophy**,** ^15^, ^16^ common brain network disruptions,^17^ and common impairments in cognitive domains.^18^, ^19^

Recently, an amyloid/tau/neurodegeneration (A/T/N) research framework has been published to advance the accurate diagnosis of AD.^20^ With this framework, AD can be identified earlier through abnormalities in amyloid and tau, even without the presence of clinical symptoms. However, molecular positron emission tomography or cerebrospinal fluid (CSF) biomarkers are often not available in the clinic, especially in the early stages for individual high‐risk patients.^21^ Therefore, researchers are constantly looking for objective biomarkers that are noninvasive and convenient to the screening of AD in its early stage. Combinations of in vivo biomarkers using the machine learning (ML) framework for predicting the progression of patients with mild cognitive impairment (MCI) to dementia has been focus of attention in recent years. Although some of these studies have achieved good predictive performance, most of the models have been validated with the leave‐one‐out approach,^22^, ^23^ or K‐fold cross‐validation loop,^24^, ^25^ which may overestimate the models’ performance. Thus, external validation of the model in an independent cohort is a crucial step in its extensive promotion.^26^

On the other hand, to our knowledge, in the field of predicting dementia conversion in LLD patients, only Lebedeva and his colleagues have used multidimensional biomarkers by employing the random forests (RF) framework of the ML method.^27^ The sensitivity of their Alzheimer's Disease Neuroimaging Initiative (ADNI) model validation in the LLD cohort was determined to be 48.0%‐65.5%, the specificity was calculated to be 62.0%‐68.6%, and the area under the curve (AUC) was 0.623‐0.737. In addition, they trained new models on the cohort of LLD patients with an AUC performance of 0.678‐0.905. However, this result is likely to be biased and overestimated by the out‐of‐bag method which was used for internally estimating the prediction performance.^28^ Therefore, a sensitive and generalizable model for identifying individuals with LOD who have a high risk of progressing to AD is lacking.

In our previous study,^29^ we chose several AD biomarkers of cognitive assessments, brain structure and function, and CSF from the ADNI cohort,^30^ to appraise their optimal sequence with the help of the event‐based probabilistic (EBP) framework. On the basis of this, we have developed the Characterizing AD Risk Events (CARE) index to measure AD progression in each individual. This CARE index model has been demonstrated to identify AD from healthy controls and accurately differentiate late MCI from early MCI.^29^ In addition, we used it to predict aMCI‐to‐AD in two longitudinal cohorts with highly accuracy, robustness, and generalization.^31^ The CARE index model works by calculating the risk scores of AD at the individual level based on the optimal temporal sequence of the biomarkers instead of the clinical diagnosis information, so it was supposed to be able to overcome the heterogeneity in the populations at high risk for AD. In this study, we applied the CARE index model to an independent aMCI population and an remitted LOD (rLOD) population to further assess its robustness and generalization and to assess whether the CARE index model can be used to monitor disease severity of the patients thereby supporting its clinical promotion.

## 2 MATERIALS AND METHODS

### 2.1 Participants

Written informed consent was obtained from all participants, and the study was approved by the responsible Human Participants Ethics Committee of the Affiliated ZhongDa Hospital, Nanjing, China. Initially, 106 elderly subjects were recruited, including 48 aMCI subjects and 58 rLOD subjects. The inclusion criteria of the aMCI subjects were proposed by Petersen et al^32^ and others,^33^ and the inclusion criteria for rLOD subjects were described previously^14^ (see S.1 in Appendix S1). The included subjects underwent follow‐up after an average of 27 months (range 16‐39 months). Twenty‐seven subjects (12 aMCI and 15 rLOD) withdrew from the study due to the development of neurological or psychiatric disease (including one rLOD subject who developed vascular dementia), withdrawal of consent, death, or relocation to other cities. Ultimately, 36 aMCI and 43 rLOD subjects were included in the present study. Of these subjects, 4 rLOD subjects and 1 aMCI subject were excluded due to head motion artefacts of more than 2.5 mm translation or 2.5° rotation, and 2 aMCI subjects were excluded for incomplete imaging coverage. The remaining 33 aMCI and 39 rLOD subjects were included in further analysis. The participants received the clinical diagnosis of possible AD with the National Institute of NINCDS‐ADRDA criteria^34^; during the follow‐up period, those patients who progressed to AD were marked as progressive subjects, while the others were marked as stable subjects. Therefore, the included participants were categorized into a progressive aMCI group (P‐aMCI) (n = 6), a stable aMCI group (S‐aMCI) (n = 27), a progressive rLOD group (P‐rLOD) (n = 10), and a stable rLOD group (S‐rLOD) (n = 29) (see Table 1). The combined high‐risk population was defined as the entire sample (N = 72) in present study, including all the aMCI subjects and rLOD subjects.

**TABLE 1:** Demographic and neuropsychological data

Items	aMCI		rLOD		*P* value		
	Stable (n = 27)	Progressive (n = 6)	Stable (n = 29)	Progressive (n = 10)	Disease	AD progression	Disease × AD progression interaction or Fisher
Age (y)	70.81 ± 4.43	72.67 ± 4.89	69.03 ± 4.95	69.6 ± 6.47	.099	.408	－
Education (y)	13.41 ± 3.16	14.00 ± 3.10	13.05 ± 3.24	11.1 ± 2.33	.075	.453	－
Sex (male:female)	18:9	2:4	10:19	5:5	－	－	.094
MMSE	27.33 ± 1.36	26.83 ± 1.84	28.31 ± 1.47	27.30 ± 1.42	.092	.078	.547
Composite Z scores of each cognitive domain							
Episodic memory	−0.33 ± 0.65	−1.05 ± 0.64	0.63 ± 0.51	−0.13 ± 0.67	<.001*	<.001*	.918
Executive function	−0.28 ± 0.64	−0.65 ± 0.61	0.46 ± 0.74	−0.19 ± 0.61	.003*	.012*	.490
Visuospatial function	−0.06 ± 0.98	−0.58 ± 1.27	0.20 ± 0.48	−0.06 ± 0.48	.061	.013	.406
Information processing speed	−0.06 ± 0.89	−0.79 ± 0.39	0.35 ± 0.91	−0.37 ± 0.91	.106	.006*	.980

Note

Values are presented as the means ± standard deviations (SD). Two‐way ANOVAs or Scheirer‐Ray‐Hare tests were applied to examine the effects of disease and AD progression on age, years of education, and neuropsychological data. Fisher's exact test was applied in the comparisons of sex.

Abbreviations: AD: Alzheimer's disease; aMCI, amnesic mild cognitive impairment; ANOVA, analysis of variance; rLOD, remitted late‐onset depression.

* *P* < .05. When comparing each cognitive domain, Bonferroni correction for multiple comparisons was performed at a significance level of *P* < .0125 (*P* = .05/4 composite scores).

### 2.2 Neuropsychological assessment

The participants completed a series of neuropsychological tests containing assessments of multiple cognitive domains, and the raw scores were Z‐transformed to composite each cognitive domain. See S.2 in Appendix S1.

### 2.3 MRI data acquisition and image preprocessing

The participants assessed at the Affiliated Brain Hospital of Nanjing Medical University (33 aMCI and 12 rLOD subjects) underwent 1.5 Tesla (T) MRI scans; the other participants assessed at the Affiliated ZhongDa Hospital (27 rLOD subjects) underwent 3.0T MRI scans. S.3 in Appendix S1 provides detailed information about image acquisition parameters and image preprocessing.

### 2.4 Individual CARE index

**FIGURE 1:**
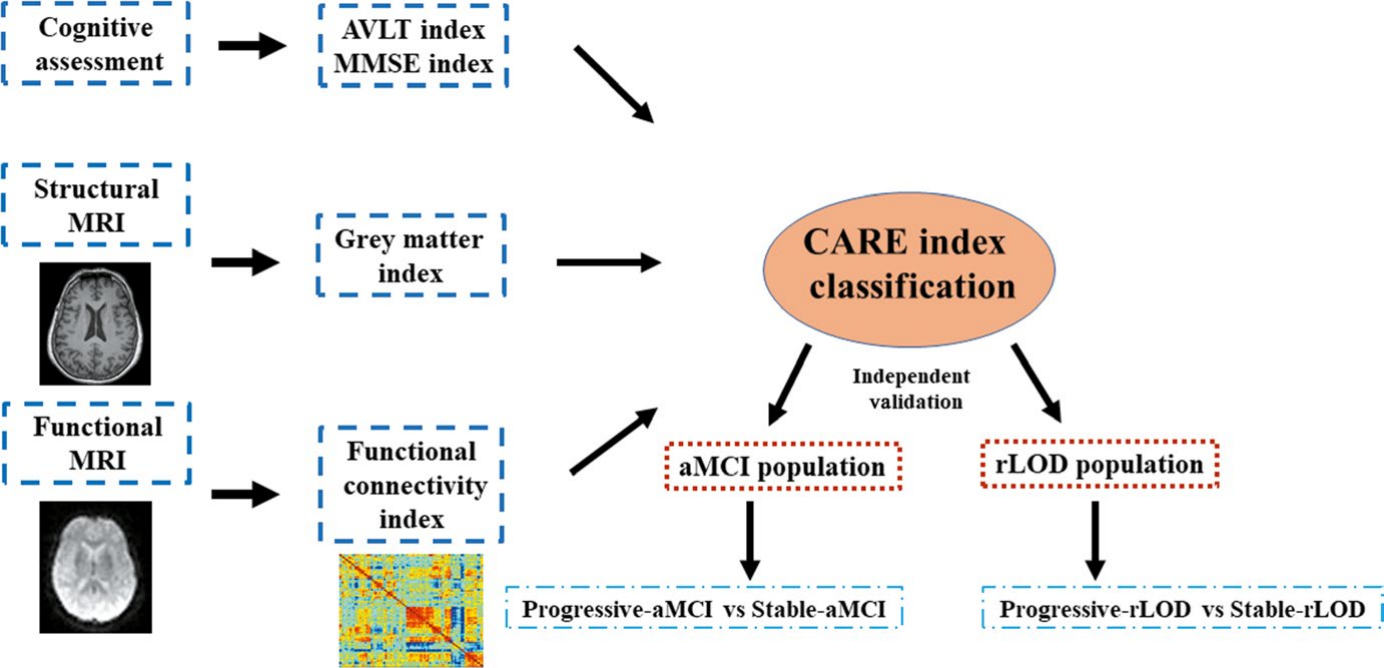
Diagram of the prediction framework and independent validation. First, the CARE index scores were calculated of the values of the selected biomarkers for each individual, which represents the subject's disease stage. Second, the CARE index scores were applied to classify stable subjects and progressive subjects in the independent aMCI and rLOD population, respectively. Notice: The CARE index model was trained on ADNI dataset in our previous study. ADNI, Alzheimer's Disease Neuroimaging Initiative

Based on our previously published research,^29^ we selected a total of 10 well‐studied AD biomarkers. Moreover, each biomarker represents an event affected by AD progression. These biomarkers consist of four levels: (a) biomarkers from brain structure: gray matter concentration indices (GMI) of the hippocampus (HIP^GMI^) and fusiform gyrus (FUS^GMI^); (b) biomarkers from brain function: functional connectivity indices (FCI) of the hippocampus (HIP^FCI^), the posterior cingulate cortex (PCC^FCI^), and the fusiform gyrus (FUS^FCI^); (c) biomarkers from cognitive assessments: Mini‐Mental State Examination (MMSE), Rey Auditory Verbal Learning Test (AVLT) immediate recall scores, and AD Assessment Scale‐Cognitive Subscale (ADAS‐Cog); (d) biomarkers from CSF: Aβ and p‐tau levels. Z‐standardization is performed to control the site effects of different magnetic field strengths.^35^ Elaborated approaches to extract FCI and GMI are presented in S.4 in Appendix S1 along with S.5. We obtained the CARE index by numbering each of the 10 biomarker events according to their optimal sequence of occurrence. Then, we obtained the CARE index score of each individual according to the maximum likelihood value of the optimal sequence (S.6‐S.9 in Appendix S1).The mathematical specifics of the optimal sequence for missing biomarker events (Aβ and p‐tau levels as well as ADAS‐Cog scores), which were not considered in the present study, are described in S.10‐S.12 in Appendix S1. The workflow is represented in Figure 1.

### 2.5 Statistical analysis

#### 2.5.1 Demographic and neuropsychological data

The statistical analyses were mainly conducted with SPSS 22.0 software (SPSS, Inc). We employed two‐way analysis of variance (ANOVA) or Scheirer‐Ray‐Hare tests with disease and AD progression as two factors for analysis of the neuropsychological and demographic data. Fisher's exact test was applied for the comparisons of sex. We mainly adopted Mann‐Whitney *U* tests to compare the differences in the CARE index scores between the P‐aMCI and the S‐aMCI subjects and between the P‐rLOD and the S‐rLOD subjects, respectively. Statistical significance was considered at *P* < .05.

#### 2.5.2 Conversion prediction

We appraised the CARE index as well as the power of individual biomarkers to differentiate progressive individuals from stable individuals using receiver operating characteristic (ROC) curves. Because there is a large difference in the number of progressive individuals and stable individuals in our study, which means the data had unbalanced labels, we introduced balanced accuracy ([sensitivity + specificity]/2) for evaluating the prediction capability of the models.^36^ In addition, we used logistic regression (LR) models in which the events were conversion to AD in the aMCI population, rLOD population, and combined high‐risk population, respectively. The independent variables were CARE index scores and demographic factors: age, sex, and years of education. Statistical significance was considered at *P* < .05. Moreover, when comparing the power of the classification of the CARE index with each single selected biomarker, we introduced the net reclassification improvement (NRI) index, which is considered to be a sensitive evaluation method to assess the improvements in the performance of the models^37^, ^38^

#### 2.5.3 The relationship between the CARE index scores and the cognitive performance

We used composite Z scores of episodic memory (EM) to reflect the severity of illness. A multi‐linear regression model was used to examine the relationship between the CARE index scores and the composite Z scores of EM at baseline in the combined high‐risk population. To further evaluate the predictive power of the CARE index scores, a multi‐linear regression model was used to investigate the relationship between the CARE index scores at baseline and the MMSE scores measured at the follow‐up in the combined high‐risk population. The variables of age, sex, and years of education were controlled in both models. The statistical threshold was set at *P* < .05.

## 3 RESULTS

### 3.1 Demographic and neuropsychological data at baseline

Table 1 suggests that there is no significant difference in age, sex, years of education or MMSE scores among the four groups of subjects. Significant main effects of disease were observed on the composite Z scores for EM, executive function (EF). The aMCI subjects exhibited lower performances on EM and EF than the rLOD subjects. Significant main effects of AD progression were found on the EM, EF, and information processing speed tests. Finally, no significant interactions of disease and AD progression in the neuropsychological data were found. Furthermore, no significant differences in follow‐up durations were observed between stable subjects and progressive subjects within each population (*Ps* > 0.05) (Table S1).

### 3.2 Discriminating stable/progressive individuals

**FIGURE 2:**
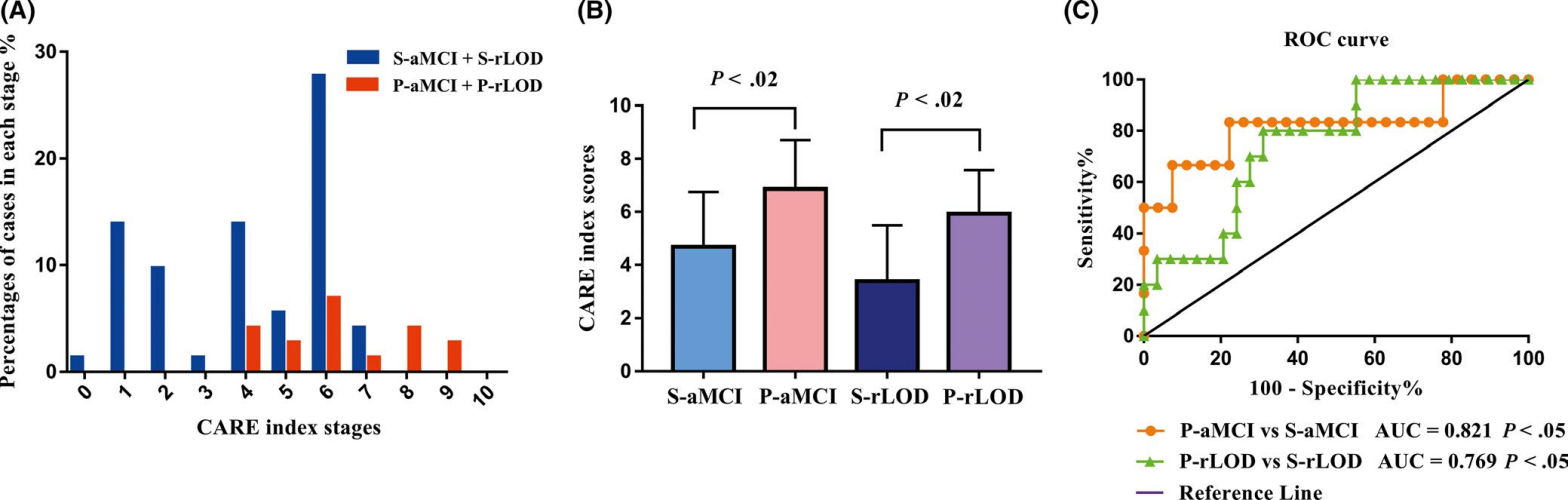
Applying the CARE index model to the independent aMCI population and rLOD population for prediction of progressive subjects and stable subjects. A, Percentages of cases in each CARE index stage at baseline in the entire sample (N = 72). Stable subjects (covering S‐aMCI and S‐rLOD) are represented in blue and progressive subjects (covering P‐aMCI and P‐rLOD) in red. B, Bars of the CARE index score differences between groups. The Mann‐Whitney *U* tests between S‐aMCI and P‐aMCI, and between S‐rLOD and P‐rLOD showed significant differences. The error bars show the standard deviation. C, The power of the ROC curve of the CARE index in identifying progressive individuals from stable individuals both in the aMCI and rLOD population at baseline. aMCI, amnesic mild cognitive impairment; AUC, area under curve; rLOD, remitted late‐onset depression; ROC, receiver operating characteristic

As shown in Figure 2, the CARE index can differentiate progressive subjects from stable subjects at baseline on an individual basis. Significant differences in the CARE index scores were noted between the P‐aMCI and the S‐aMCI subjects and between the P‐rLOD and the S‐rLOD subjects (*Ps* < 0.05). AD diagnoses were predicted in the aMCI population with a balanced accuracy of 80.6%, a sensitivity of 83.3%, a specificity of 77.8%, and an AUC of 0.821. They were also predicted in the rLOD population with a balanced accuracy of 74.5%, a sensitivity of 80.0%, a specificity of 69.0%, and an AUC of 0.769 (Figure 2C and Table 2). Hazard ratio (HR) and statistical significance of each variable in the LR models are listed in Table 3. Goodness of fit of the logistic models were evaluated by the Hosmer‐Lemeshow tests (*Ps* > 0.05).Increasing CARE index was significant hazard for AD conversion of individual patients in the aMCI population (HR = 2.642, 95% confidence interval (CI) = 1.035‐6.743), rLOD population (HR = 2.143, 95% CI = 1.125‐4.081), and combined high‐risk population (HR = 1.983, 95% CI = 1.243‐3.164).

**TABLE 2:** Classification results of CARE index and each selected biomarker indices in aMCI population and rLOD population separately

Predictors	AUC (95% CI)	*P* value	Sensitivity%	Specificity%	Balanced Accuracy%	NRI* Estimate
aMCI population						
CARE index	0.821 (0.649‐0.932)	.012	83.3	77.8	80.6	‐
MMSE	0.608 (0.423‐0.773)	.453	33.3	92.6	63.0	−0.352
AVLT	0.778 (0.600‐0.903)	.054	83.3	77.8	80.6	0.000
HIP^FCI^	0.673 (0.488‐0.825)	.094	66.7	77.8	72.2	−0.167
PCC^FCI^	0.710 (0.526‐0.854)	.021	100.0	59.3	79.6	−0.019
FG^FCI^	0.722 (0.539‐0.863)	.050	100.0	48.2	74.1	−0.130
HIP^GMI^	0.765 (0.586‐0.895)	.006	83.3	70.4	76.9	−0.074
FG^GMI^	0.728 (0.546‐0.868)	.041	66.7	74.1	70.4	−0.204
rLOD population						
CARE index	0.769(0.606‐0.888)	.001	80.0	69.0	74.5	‐
MMSE	0.695 (0.527 −0.832)	.032	100.0	27.6	63.8	−0.214
AVLT	0.736 (0.571‐0.864)	.027	50.0	96.6	73.3	−0.024
HIP^FCI^	0.693 (0.525‐0.830)	.024	90.0	55.2	72.6	−0.038
PCC^FCI^	0.745(0.580‐0.871)	.002	90.0	55.2	72.6	−0.038
FG^FCI^	0.593(0.424‐0.747)	.387	50.0	72.4	61.2	−0.266
HIP^GMI^	0.617 (0.448‐0.768)	.266	60.0	65.5	62.8	−0.235
FG^GMI^	0.562(0.394‐0.720)	.609	40.0	89.7	64.8	−0.193

Abbreviations: aMCI, amnesic mild cognitive impairment; AUC, area under the receiver‐operator curve; AVLT, Rey Auditory Verbal Learning Test; CARE, characterizing AD risk event; FCI, functional connectivity indices; FG, fusiform gyrus; GMI, gray matter indices; HIP, hippocampus; MMSE, Mini‐Mental State Examination; PCC, posterior cingulate cortex; rLOD, remitted late‐onset depression.

* NRI was used to compare the seven selected biomarker models to the CARE index model.

**TABLE 3:** HRs with 95% CIs for conversion from aMCI and rLOD to AD obtained by LR models separately

	Hazard ratio (CI)	*P* value	*β*
aMCI (n = 33) to AD			
CARE index	2.642 (1.035‐6.743)	.042*	0.972
Age	1.189 (0.898‐1.574)	.228	0.173
Education	1.080 (0.762‐1.531)	.666	0.077
Male	0.181 (0.016‐2.063)	.169	−1.708
rLOD (n = 39) to AD			
CARE index	2.143 (1.125‐4.081)	.020*	0.762
Age	0.961 (0.794‐1.162)	.682	−0.040
Education	0.881 (0.646‐1.200)	.908	−0.127
Male	1.864 (0.317‐10.980)	.491	0.623
Combined high‐risk population (N = 72) to AD			
CARE index	1.983 (1.243‐3.164)	.004*	0.685
Age	1.010 (0.886‐1.150)	.886	0.010
Education	0.910 (0.744‐1.114)	.360	−0.094
Male	0.733 (0.207‐2.597)	.630	−0.311

Abbreviations: AD, Alzheimer disease; aMCI, amnesic mild cognitive impairment; LR, logistic regression; rLOD, remitted late‐onset depression.

* *P* < .05.

### 3.3 Robustness and power of the CARE index compared to a single biomarker

As explained in Table 2, the CARE index outperforms each of the selected biomarkers in distinguishing the progressive and stable subjects either in the aMCI or in rLOD populations. Although the balanced accuracy of AVLT scores is high in the aMCI population, its AUC is not as high as that of the CARE index. In addition, AVLT scores did not show high generalization and stability across datasets in our previous study.^31^ Besides, the sensitivity of AVLT scores at the optimal cutoff is low in the rLOD population.

### 3.4 Behavioral significance of the CARE index measured at baseline

**FIGURE 3:**
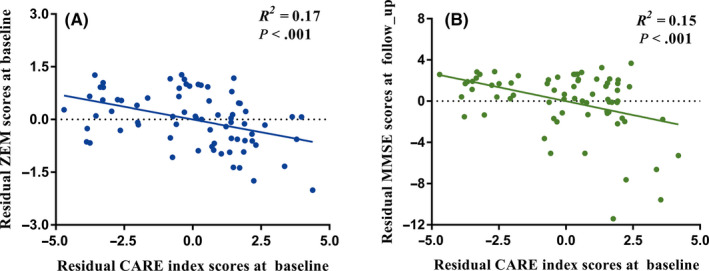
The correlations between the CARE index and cognitive performance in the combined high‐risk population (N = 72). A, Significant correlation between the baseline CARE index scores and ZEM measured at baseline (*P* < .001). B, Significant correlation between the baseline CARE index scores and MMSE scores measured at mean follow‐up period of 27 months (*P* < .001). Note: One outlier of MMSE score that was detected using Grubbs' test has been excluded in this analysis. MMSE, Mini‐Mental State Examination; ZEM: composite Z scores of episodic memory

As illustrated in Figure 3A, in the combined high‐risk population, a negative correlation was detected between the CARE index scores and the composite Z scores of the EM at baseline (*R* ^2^  = .17, *P* < .001). The higher the CARE index score is, the lower the composite Z scores of the EM and the more severe the disease.

### 3.5 Predictive value of the CARE index scores measured at baseline

As illustrated in Figure 3B, in the combined high‐risk population, a negative correlation was observed between the CARE index scores at baseline and MMSE scores at follow‐up (*R* ^2^ = .15, *P* < .001). During the follow‐up period of 27 months, the CARE index score was inversely proportional to the global cognition performance.

## 4 DISCUSSION

In the present study, we demonstrated that the CARE index model can effectively differentiate individuals progressing to AD from stable individuals in both the MCI population and the rLOD population with satisfactory generalization. In addition, the CARE index demonstrated better performance than each single collected biomarker in our study. Besides, the CARE index scores can be used to monitor disease severity, and the scores at baseline can be used to predict the long‐term performance of high‐risk patients.

In this study, the CARE index model was applied to the aMCI population assessed using 1.5T MRI scanners with high robustness. Prior to this study, we validated the CARE index model in an independent Nanjing Aging and Dementia Study cohort with 3.0T MRI scanners with an AUC of 0.861, a sensitivity of 81.3%, a specificity of 90.0%, and a balanced accuracy of 85.7%.^31^ These data show that the model has satisfactory generalizability and robustness in independent cohorts, even in different MRI scan modes. Furthermore, there are several studies comparable to our study that demonstrates generalizability. Lebedeva and colleagues^39^ also applied their prediction model employing the RF framework trained on the ADNI cohort to an independent AddNeuroMed^40^ cohort. They reported a sensitivity of only 78.0% when they predicted the conversion of MCI patients with a 1‐year follow‐up. In our study, the CARE index model achieved better sensitivity of the independent validation with mean follow‐up period of 24 months in the aMCI population. The period of follow‐up in their study is shorter than 20 months while a considerable number of MCI cases may convert to AD later,^41^ which will reduce the positive predictive value of classification results.^42^ Hall et al^43^ evaluated how well a disease state index (DSI) model generalized to 4 different cohorts: DESCRIPA,^44^ ADNI, AddNeuroMed, and the Kuopio MCI study.^45^ However, the performance of the model was not sufficiently robust and stable, when they combined other independent cohorts as a training dataset with an AUC of 0.72‐0.76 and an accuracy of 0.67‐0.79. In a recent novel study, Spasov, S and his colleagues^46^ developed a deep learning approach of structural MRI images, demographic, neuropsychological, and *APOE* ε4 data. They used the algorithm to identify MCI patients progressing to AD within 3 years from among stable MCI patients with an AUC of 0.925, a sensitivity of 87.5%, a specificity of 85%, and an outer 10‐fold cross‐validated accuracy of 86%. Thus, this model was not validated in a completely independent dataset. In addition, they did not explore the relationship between behavior and the predictive indicators, so it is difficult to understand the pathophysiological implications of the predictive indicators for clinicians.

Another principal novelty of this study was that the CARE index model can effectively identify progressive rLOD patients from stable rLOD patients. It is worth noting that it is the first time applied the model based on EBP framework to LOD population. As we mentioned, only Lebedeva and colleagues have used an RF framework to predict dementia conversion in LLD patients.^27^ In our study, the AUC, sensitivity, and specificity of the CARE index model were better than those values in their research when they validated the ADNI model in the LLD population. In addition, the subjects in their study included both early‐onset depression (EOD) and LOD. EOD is likely to denote an independent risk factor that predisposes patients to dementia rather than a prodrome of AD.^11^ We also noted that the specificity of the CARE index was not high enough in the rLOD population. We inferred that the reason was that, except for the common biomarkers of AD progression with MCI, some biomarkers may be more specifically related to the conversion of rLOD to AD, such as EF,^47^ functional connectivity of the amygdala,^48^ and white matter damage in the middle anterior corpus callosum.^27^ However, sensitivity is a key indicator for distinguishing potential AD patients for early intervention and is more crucial than specificity. In addition, it is important that our result comes from an independent validation of the model. Therefore, the CARE index may still be considered a good risk‐screening tool to identify progressive individuals in the rLOD population.

We believe that the main causes for the better robustness and generalization of the CARE index model are as follows. First, in our model, the biomarkers that we selected have been well established in studies on the conversion to AD. Numerous studies have confirmed AVLT scores as a reliable indicator related to the conversion of MCI to AD.^49^, ^50^ Meanwhile, the rLOD group of patients had noticeably poorer performance in the AVLT scores than the healthy group.^51^ Many studies have been conducted on the abnormalities of the structure and function of the hippocampus associated with conversion to AD in both MCI^52^ and LOD patients.^53^, ^54^ The PCC was thought to be a structural and functional core hub of the default mode network (DMN), and the DMN abnormalities have been associated with AD disease progression.^55^ In addition, according to previous studies, the DMN was considered the neural foundation of the link between AD and LOD.^6^ Structural and functional indicators of the fusiform gyrus were also incorporated into the CARE index model. Young and colleagues found that brain atrophy in particular regions occurred in a distinct sequence in the AD spectrum; in particular, the hippocampus and entorhinal cortex are initially affected with a progression to the fusiform gyrus and the middle temporal gyrus.^56^ The functional connectivity of fusiform gyrus has also been thought to be a part of the DMN.^57^ It is interesting that in a task‐based fMRI study, the hippocampus and fusiform gyrus were the important brain regions associated with cognitive decline in LLD patients.^58^ Second, we believe that the methodological advantage is the reason why the CARE index achieved good performance while overcoming disease heterogeneity. The pathogenesis of AD as a clinical entity is a series of pathophysiology‐related incidents associated with neurodegeneration and amyloidosis. The CARE index model mainly studies a series of temporally dependent processes of biomarker events during AD development, instead of the clinical diagnostic information.^29^ In addition, the CARE index scores were calculated to measure the disease stage at the individual level rather than describing the nature of the whole cohort. The results of our application are very meaningful. It proved that LOD, aMCI, and AD represent a continuous spectrum from another perspective. Currently, open databases with LOD patients are not large enough for ML, and it is suggested that the model trained in the databases for MCI and AD studies (ADNI, AddNeuroMed, etc) also has the prospect of distinguishing progressive individuals with LOD.

As demonstrated in Table 2, the CARE index was superior to each of the selected biomarker indices in distinguishing progressive individuals from stable individuals. This suggests that the CARE index can effectively integrate information contained in different modal biomarkers of AD progression. Furthermore, a negative predictive relationship was observed between CARE index scores and the composite Z scores of EM, which have been considered a main clinical feature of AD. The results from the LR models also showed that an increase in the CARE index was a significant hazard for conversion in individual high‐risk patients. Consistent with our previous view,^29^ the CARE index model mainly emphasizes the temporally dependent process of a series of pathophysiological events in AD development. Relying on this advantage, it links the existence of any specific biomarker for AD in the context of the entire progression of the disease. Therefore, it could be used to monitor disease progression as an AD staging system for individual high‐risk patients. In addition, a negative predictive relationship was observed between CARE index scores measured at baseline and MMSE scores measured at the follow‐up, which also proves that the CARE index is a good predictor for the progression of AD.

In the present study, the CARE index model was found to be replicable and accurate for identifying patients with a high risk of progressing to AD. Additionally, this model based on the EBP framework was found not to be limited by the heterogeneity of the population, which will greatly facilitate its application. In addition, this model also has the advantages of being noninvasive, convenient, and especially capable of monitoring patients’ clinic progression, which is essential for clinicians. In summary, this model is potentially important and clinically valuable.

### 4.1 Limitations

First, the sample size in our study was relatively small, particularly in the progressive groups. Second, the MRI scanners used in the rLOD group displayed varied magnetic field strengths. However, z‐standardization is performed to control for the site effects of different magnetic field strengths. Third, although our study showed that the CARE index can well overcome the disease heterogeneity, further studies should be considered to incorporate more reliable biomarkers, such as EF and diffusion tensor imaging, to improve the performance of the model.

## 5 CONCLUSION

In conclusion, the CARE index model was successfully used to predict AD in an independent aMCI population and an rLOD population with satisfactory generalizability and balanced accuracy. In particular, the CARE index model should be used to monitor patients’ clinical progression. Our findings suggest that the CARE index can be effectively applied to detect individual high‐risk patients in clinical practice to develop early treatment strategies for preventing AD progression.

## CONFLICT OF INTEREST

The authors declare that they have no conflict of interest.

## Supporting information

Appendix S1
