# Supplementary material for: Predicting conversion to Alzheimer's disease among individual high‐risk patients using the Characterizing AD Risk Events index model
Source: CNS Neurosci Ther. 2020 Apr 3;26(7):720–9. doi: 10.1111/cns.13371 (PMC7298996; doi:10.1111/cns.13371)
Supplement: Supplementary file 1 — Appendix S1 [file CNS-26-720-s001.docx]

# Supplementary Methods

**S.1 Inclusion and exclusion criteria**

All aMCI subjects met the diagnostic criteria proposed by Petersen et al. (1999) and others (Winblad et al., 2004): including 1) subjective memory impairment corroborated by the subject and an informant, 2) objective memory performances documented by an AVLT-DR score ≤ 1.5 standard deviations of age-adjusted and education-adjusted norms (the cut-off was ≤ 4 correct responses on 12 items for ≥ 8 years of education), 3) normal general cognitive function evaluated by an MMSE score ≥ 24, 4) a CDR of 0.5, with at least a 0.5 in the memory domain, 5) minimal or no impairment of routine daily life activities, 6) the absence of dementia or insufficiency in meeting the National Institute of Neurological and Communicative Disorders and Stroke and the AD and Related Disorders Association (NINCDS-ADRDA) and DSM-IV criteria for AD. Exclusion criteria were as follows: 1) a history of stroke (modified Hachinski score of > 4), alcoholism, head injury, Parkinson’s disease, epilepsy, major depression (excluded by a self-rating depression scale), or other neurological or psychiatric illness (excluded by clinical assessment and case history), 2) major medical illness (e.g., cancer, anaemia, and thyroid dysfunction), 3) severe visual or hearing loss, 4) T2-weighted MRI showing major white matter changes, infarction, or other lesions (two experienced radiologists executed the scans).

The inclusion criteria for rLOD subjects were as follows: (1) they had previously met the criteria for major depressive disorder in DSM-IV and had remitted for > 6 months before enrollment; (2) the age was > 60 years;(3) the age at first depression onset was > 50 years; (4) Hamilton Depression Rating Scale scores < 7, and Mini-Mental State Examination (MMSE) scores > 24; (5) duration of illness < 5 years and a medication-free period > 3 months prior to the assessment; (6) absence of other major psychiatric disorders, including abuse of or dependence on psychoactive substances; (7) absence of primary neurological disorders, including dementia or stroke; (8) absence of a medical illness that impairs cognitive function; (9) no history of electroconvulsive therapy; and (10) no gross structural abnormalities on T1-weighted images, and no major white matter changes such as infarction or other vascular lesions on T2-weighted MRI. Exclusion criteria were as follows: (1) another major psychiatric illness, including substance abuse or dependence; (2) primary neurological illness, including dementia or stroke; (3) medical illness impairing cognitive function; (4) history of electroconvulsive therapy; (5) T2-weighted MRI showing major white matter (WM) changes, infarction, or other lesions (two experienced radiologists analyzed the scans).

S.2 Demographic and neuropsychological data

The participants completed a series of neuropsychological tests containing assessments of multiple cognitive domains and consisted of the Mini-Mental State Examination (MMSE); Auditory Verbal Learning Test–immediate recall (AVLT-IR); Auditory Verbal Learning Test–5-min delayed recall (AVLT-5-min-DR); Auditory Verbal Learning Test–20-min delayed recall (AVLT-20-min-DR); Rey-Osterrieth Complex Figure Test (ROCFT); Rey-Osterrieth Complex Figure Test–20-min delayed recall (ROCFT-20-min-DR); Trail-Making Tests A and B (TMT-A and B); Symbol Digit Modalities Test (SDMT); verbal fluency test (VFT); and clock drawing test (CDT). We divided these tests into global cognitive function (one test: MMSE) and four cognitive domains: episodic memory (EM) (two tests: AVLT–20-min-DR and ROCFT–20-min-DR), information processing speed (IPS) (two tests: SDMT and TMT-A), visuospatial function (VF) (two tests: ROCFT and CDT), and executive function (EF) (two tests: VFT and TMT-B). Composite Z scores were used to reflect the performance for each cognitive domain. Notably, for tests measured by timing, including TMT-A, TMT-B, the raw scores were defined as the reciprocal of the time for the tests.To increase statistical power by reducing random variability, this study composited the neuropsychological tests into four cognitive domains and transformed the raw scores into four composite Z scores. The following is the equation for Z transformation: where is the Z score of the *i*th subject, r_i_ is the raw score of the *i*th subject, r is the average raw score of the neuropsychological test for all subjects, and *S* is the standard deviation of the scores.

$$Z_{i}=\frac{r_{i}-\bar{r}}{S}$$

Bonferroni correction for multiple comparisons was performed at a significance level of *p* < 0.0125 (*p* = 0.05/4 composite scores). In addition, when calculating the CARE index scores, we standardized the tests in the present research into the ADNI dataset. Given the cognitive measure scores between different datasets, they may have different test components.

S3. Image acquisition details and image pre-processing

The Participants who were recruited at the Affiliated Brain Hospital of Nanjing Medical University(33 aMCI and 12 rLOD subjects) underwent fMRI scans by 1.5 Tesla General Electric (General Electric Medical Systems, Milwaukee, Wisconsin) scanner; the other participants who were recruited at the Affiliated ZhongDa Hospital (27 rLOD subjects) underwent fMRI scans by 3.0 Tesla Verio Siemens (Erlangen, Germany) scanner .The details are as follows: (i) 1.5 Tesla MRI scanner: Axial R-fMRI data (no cognitive tasks were performed, eyes were closed, and ears were occluded) were obtained with a single-shot gradient-recalled echoplanar imaging sequence: repetition time (TR) = 3000 ms; echo time( TE) = 40 ms; flip angle (FA) = 90°; acquisition matrix = 64 × 64; field of view(FOV) = 240 mm × 240 mm; thickness = 4.0 mm; gap = 0 mm; and 3.75 mm × 3.75 mm in-plane resolution. The R-fMRI scan lasted 7 min and 6 s. High-resolution T1-weighted axial images covering the whole brain were acquired using a 3D spoiled gradient echo sequence shown as follows: repetition time (TR) = 9.9 ms, echo time (TE) = 2.1 ms, flip angle = 15°, acquisition matrix = 256 × 192, field of view = 240 mm× 240 mm, thickness = 2.0 mm, gap = 0 mm, and number of excitation (NEX) = 1.0. (ii)3.0 Tesla MRI scanner:Axial R-fMRI data (no cognitive tasks were performed, eyes were closed, and ears were occluded) were obtained with TR = 2000 ms, TE = 25 ms, FA= 90°,acquisition matrix = 64 × 64, FOV = 240 mm × 240 mm, thickness = 4.0 mm, gap = 0 mm, and 3.75 mm × 3.75 mm in-plane resolution，number of slices = 36, and voxel size = 3.75 × 3.75 × 4mm^3^.A rapid gradient echo sequence was prepared using three-dimensional magnetization to obtain high resolution T1-weighted anatomical images as follows: TR = 1900 ms, TE = 2.48ms; FA= 9°, acquisition matrix = 256 × 256, FOV = 250× 250 mm, thickness = 1.0 mm, gap = 0 mm, number of slices = 176, and voxel size = 1 × 1 × 1 mm^3^.Additionally, routine axial T2-weighted images were acquired to rule out subjects with major WM changes, cerebral infarction or other lesions using flair sequence as below: TR = 8400 ms, TE = 94 ms, FA= 150°,acquisition matrix = 256 × 256, FOV = 230 ×2 30mm, thickness = 5.0 mm, gap = 0 mm, and number of slices = 20.

Image pre-processing:Image data pre-processing was performed using the Statistical Parametric Mapping 8 (SPM8)package (Institute of Neurology, London, UK) and the Data Processing & Analysis for Brain Imaging (DPABI V3.1)^40^ toolbox operating on the MATLAB platform (The MathWorks, Inc., Natick, MA, USA). We discarded the first ten volumes and corrected the remaining images for timing diversifications and motion effects. According to the Montreal Neurological Institute template, we normalized the obtained images in a spatial manner in the identical stereotactic space using 12 affine transformations and then resampled them to 4 × 4 × 4 mm^3^ voxels. Subsequent pre-processing covered linear detrending and temporal band-pass filtering (0.015–0.1 Hz). The main purpose was to reduce the impact of high-frequency physiological noise and low-frequency drift. Last, we regressed out several nuisance variables, including 24-dimensional head-motion parameters as well as the CSF signal from the data.

S4. Functional connectivity indices of the regions of interest

This study calculated the functional connectivity indices (FCI) of the three regions of interest (ROIs): bilateral hippocampus (HIP^FCI^), posterior cingulate cortex (PCC^FCI^), and fusiform gyrus (FUS^FCI^). The details regarding the FCI calculation stream were found in our previously published study ([G. Chen et al., 2016](#_ENREF_1)). First, the whole cerebral cortex was separated into 90 regions based on the Automated Anatomical Labeling (AAL) template, and the blood oxygen level dependent (BOLD) time series of each region was extracted using the AAL template mask from the preprocessed resting-state dataset ([Tzourio-Mazoyer et al., 2002](#_ENREF_6)). Second, functional connectivity between each ROI and the other brain regions was calculated using the Pearson cross-correlation analysis. Thus, a vector consisting of 89 cross-correlation coefficient (CC) values for each ROI was obtained. Finally, each ROI’s FCI value - identified separately as HIP^FCI^, PCC^FCI^, and FUS^FCI^ - was calculated by summating 89 CC values within each ROI’s vector and averaging them across each pair of bilateral ROIs.

*i* = k+1

S5. Gray matter index

For each subject, the gray matter index ([Xie et al., 2009](#_ENREF_8)) of each brain region (using the same AAL template) was calculated using SPM8 software (www.fil.ion.ucl.ac.uk/spm/software/spm8/). First, the anatomical image of each individual’s brain was normalized into the Montreal Neurological Institute (MNI) space. Second, the gray matter of the whole brain was segmented and separated from WM and cerebrospinal fluid (CSF) areas, and a threshold of 0.8 was used to exclude non-gray-matter areas. Third, each region’s gray matter concentration index was determined by summing the gray matter concentration values of all voxels within the region and averaging across each pair of bilateral ROIs.

S6. Event-based probabilistic model

Given that a set of N events, E_1_, E_2_, …, E_N_, is measured by N biomarkers, x_1_, x_2_, …, x_N_, respectively, the temporal order of events, S={s(1), s(2), …, s(N)}, is calculated by a permutation of the integers 1, …, N. For subjects j=1, …, J, the dataset X could be regarded as X={X_1_, X_2_, …, X_J_}. Specifically, X_J_ represents the subject j data that is given by X_j_={x_1j_, x_2j_, …, x_Nj_}, where x_ij_ is the ith biomarker measurement for subject j. This study determined the optimal temporal order in a data-driven manner, based on the criteria that the optimal temporal order, defined as the S^optimal^, yielded the highest probability in measuring dataset X. That is, the p(X|S) value of the S^optimal^ sequence was calculated to be maximal among all of the possible sequences. To accomplish this objective, we first estimated the likelihood of measurement x_ij_ given that biomarker event E_i_ has or has not occurred. These likelihoods are labeled below:

$p(x_{ij}\left| E_{i})=likelihood of measurement x_{ij} given that event E_{i} has occurred \right.$ (1)
$p(x_{ij}\left| \neg E_{i})=likelihood of measurement x_{ij} given that event E_{i}\mathrm{has}not \mathrm{occurred} \right.$ (2)

We assumed that subject j is at stage k, although the authentic biomarkers sequence and the subject’s stage were unavailable. This means that for subject j, events E_s(1)_, E_s(2)_, …, E_s(k)_ already have occurred, and events E_s(k+1)_, E_s(k+2)_, …, E_s(N)_ have not occurred. The likelihood of data X_j_ given the sequence S and the subject’s stage at k was obtained using the formula below:

$p\left( X_{j} | S,k \right)= \prod_{i=1}^{k} p\left( x_{ij} | E_{s\left( i \right)} \right)\prod_{i=k+1}^{N} p\left( x_{ij} | {\neg E}_{s\left( i \right)} \right)$ (3)

Where $\prod_{i=1}^{k} p\left( x_{ij} | E_{s\left( i \right)} \right)$ is the overall likelihood of measurements given that corresponding events have already occurred, $\prod_{i=k+1}^{N} p\left( x_{ij} | {\neg E}_{s\left( i \right)} \right)$ is the overall likelihood of measurements given that these events have not yet occurred. Then, we obtained the likelihood of data X_j_ in the condition of sequence S by summing the likelihood values of data X_j_ across all possible stages within sequence S, as shown in equation (4) below:

$p\left( X_{j} | S \right)= \sum_{k=0}^{N} p\left( k \right)p\left( X_{j} | S,k \right)$ (4)

Next, we combined the measurements of all subjects, j=1, …, J, assuming that the intersubject relationship is independent:

$$p\left( X | S \right)=\prod_{j=1}^{J} p\left( X_{j} | S \right)=\prod_{j=1}^{J} \sum_{k=0}^{N} p\left( k \right)p\left( X_{j} | S,k \right)$$

$=\prod_{j=1}^{J} \sum_{k=0}^{N} p\left( k \right)\left[ \prod_{i=1}^{k} p\left( x_{ij} | E_{s\left( i \right)} \right) \prod_{i=k+1}^{N} p\left( x_{ij} | {\neg E}_{s\left( i \right)} \right) \right]$ (5)

In theory, the above analysis needs to be repeated for each possible sequence to determine the sequence S^optimal^ with the maximal value of $p\left( X | S \right)$. However, such a computation strategy is extremely time consuming; total calculation times in this study would be 2.7942e+009, given that there are 10 biomarker events, 11 possible stages (including stage 0), and 70 subjects (cognitively normal [CN] and AD groups from ADNI2 dataset only，details see our previous study ([G. Chen et al., 2016](#_ENREF_1)) involved). Therefore, we employed a greedy algorithm to improve processing efficiency.

S7. Event occurrence and nonoccurrence distribution modeling

We used a mixture model of two Gaussian distributions to fit the event data from the CN and AD groups, based on the assumption that an event occurring and an event not occurring are estimated by a mixed distribution of normal and abnormal groups. The fitted Gaussian distributions separated the data into two groups, i.e., abnormal (event occurred) and normal (event did not occur), similar to the approach by Young et al. ([Young et al., 2014](#_ENREF_9)). Notably, we modified Young et al.’s approach by applying a k-mean clustering algorithm to separate the whole distribution into two clusters before applying the Gaussian mixture model fitting. This modified modeling method led to high consistency in the obtained model.

S8. Self-growing greedy algorithm

The amount of time such an analysis would take to find a global optimal result is unpredictable due to the randomized initial sequence, and it may be quite long due to the inevitable searching loop. Therefore, we developed a new greedy algorithm to address this deficiency. The greedy algorithm explores the globally optimal solution by making the locally optimal choice at each stage, in a greedy heuristic manner. The greedy Markov chain Monte Carlo (MCMC) algorithm is a useful approach to find globally optimal results. Specifically, we started with a set of all possible initial root sequences, each of which consisted of two randomly selected events from the 10 biomarker events total. Second, for each initial sequence S, we generated the children of S by inserting a randomly selected event from the remaining events. Third, we selected the children sequence with the maximal $p\left( X | S \right)$ value; this replaced the initial sequence. Then, we entered another randomly selected event into the sequence and repeated the second and the third steps until no events were left. Thus, we generated whole sequences for each root sequence. Ultimately, we determined the sequence with the maximum $p\left( X | S \right)$ value as the final optimal sequence, S^optimal^. We repeated this greedy algorithm 100 times to ensure the S^optimal^ had a high reliability. 45 CN and 25 AD subjects from ADNI2 were used to determine the S^optimal^ before , details see our previous study ([G. Chen et al., 2016](#_ENREF_1)). Note that the aMCI and rLOD subjects in this study were not used to train the S^optimal^. The S^optimal^ reflects the order in which sequential pathophysiological events occurred and provides a numeric score to measure disease progression from one stage to the next.

S9. The CARE index score calculation based on the obtained sequence

Using the following equation to determine each subject’s CARE index score, we calculated the likelihood value of *k* at each possible stage in the sequence and defined the CARE index score as that at which *k* had the highest likelihood value at the S^optimal^:

${argmax}_{k}P\left( k \right)= \prod_{i=1}^{k} p\left( x_{ij} | E_{S^{optimal}\left( i \right)} \right) \prod_{i=k+1}^{N} p\left( x_{ij} | {\neg E}_{S^{optimal}\left( i \right)} \right)$ (6)

In equation 6, implications of $\prod_{i=1}^{k} p\left( x_{ij} | E_{S^{optimal}\left( i \right)} \right)$ and $\prod_{i=k+1}^{N} p\left( x_{ij} | {\neg E}_{S^{optimal}\left( i \right)} \right)$ refer to those in equation 3, except that the optimal sequence, S^optimal^, is obtained.

Mathematical detail of missing biomarkers

S10.Biomarker events

Ten well-studied AD biomarkers, as described above, were selected (Table S1); each represents an event that occurs along with AD progression.

Table S0. The order of the 10 progressive events in AD development represented by the 10 well-studied biomarkers.

| E1 | E2 | E3 | E4 | E5 | E6 | E7 | E8 | E9 | E10 |
| --- | --- | --- | --- | --- | --- | --- | --- | --- | --- |
| HIP^FCI^ | PCC^FCI^ | Aβ1-42 | p-tau | MMSE | ADAS-Cog | AVLT | HIP^GMI^ | FUS^GMI^ | FUS^FCI^ |

S11.Weighted average stage

The mathematical detail of event-based probabilistic (EBP) model is described earlier ([G. Chen et al., 2016](#_ENREF_1)，J. Chen et al.,2019). The EBP model determines the optimal order of biomarker events (i.e., S^optimal^ = { E1, E2, E3, E4, E5, E6, E7, E8, E9, E10}). It also determines the likelihood of subject j being in stage k, given the biomarker measurement X_j_ and S^optimal^, by the formula below:

$p\left( X | S,k \right)=\left[ \prod_{i=1}^{k} p\left( x_{ij} | E_{s\left( i \right)} \right) \prod_{i=k+1}^{N} p\left( x_{ij} | {\neg E}_{s\left( i \right)} \right) \right]$ (7)

where Xj= {x1j, x2j, …, xNj}, and x_ij_ is the ith biomarker measurement for subject j. The normalized likelihood is defined as:

$p_{norm}\left( X_{j} | S,k \right)=A_{j}*p\left( X_{j} | S,k \right),$(8)

where normalization factor is determined by:

$\sum_{k=0}^{N} p_{norm}\left( X_{j} | S,k \right)= A_{j}* \sum_{k=0}^{N} p \left( X_{j} | S,k \right)= 1$(9)

The weighted average (WA) stage *k* *_j, weighted average_* for subject j is defined as

$k_{j, weighted average}= \sum_{k=0}^{N} {k*p}_{norm}\left( X_{j} | S,k \right)$ (10)

S.12 Soptimal in missing biomarkers

In the previous EBP model ([G. Chen et al., 2016](#_ENREF_1))., the individual subject's disease stage was determined by the “winner take all” approach, i.e., the stage k with the highest likelihood value determines the subject's disease stage. However, this poses a potential problem when a biomarker is missing. For example, when a subject's disease stage corresponds to the missing marker k, it is impossible to determine the subject's disease stage to be k in the “winner take all” approach. The disease stage will fall to the next available highest likelihood stage, most likely k−1 or k+1. To address this problem, here we employ the WA stage defined in Equ (10). Theoretically, the WA stage of a subject can be k even when the corresponding marker is missing; this is demonstrated with a representative subject in Fig. S1. In the case of missing biomarker *i_missing_* , $p\left( x_{i missing j} | E_{s(i missing)} \right)$ and $p\left( x_{i missing j} | {\neg E}_{s(i missing)} \right)$ were set to be 1. This is equivalent to removing them from Equ. (7) without having to modify the existing programs. Meanwhile, for $k=i_{missing},p\left( X_{j} | S,k \right)$ was set to 0. With this numerical modification, neither the analytical equations (7–10) nor the existing programs from [6] needed to be modified. However, for clarity, Equ. (7) was rewritten to account for missing biomarker(s):

$$p\left( X_{j} | S,k \right) = 0, k = i_{missing}$$

$$p\left( X_{j} | S,k \right)=\left[ \prod_{i=1}^{k} p\left( x_{ij} | E_{s\left( i \right)} \right) \prod_{i=k+1}^{N} p\left( x_{ij} | {\neg E}_{s\left( i \right)} \right) \right] , i {\neq i}_{missing}$$

The program was employed to calculate optimal when the biomarkers were missing. The WA stage for each subject was calculated.

**Table S1**

| **Table S1. Follow-up durations.** | | | | | |
| --- | --- | --- | --- | --- | --- |
| **Items** | **aMCI** | | **Z value** | ***p* value** |  |
|  | **Stable (*n* = 27)** | **Progressive (*n* = 6)** |  |  |  |
| Range of follow-up (months) | 16 - 34 | 18 - 34 |  |  |  |
| Length of follow-up (months) | 23.78 ± 4.20 | 22.83 ± 5.90 | 0.726 | 0.468 |  |
|  | **rLOD** | | **Z value** | ***p* value** |  |
|  | **Stable (*n* = 29)** | **Progressive (*n* = 10)** |  |  |  |
| Range of follow-up (months) | 21.5 - 39 | 22 - 37 |  |  |  |
| Length of follow-up (months) | 30.29 ± 4.24 | 29.00 ± 5.10 | 0.660 | 0.509 |  |
|  | **Combined high-risk population** | | **t value** | ***p* value** |  |
|  | **Stable (*n* = 56)** | **Progressive (*n* = 16)** |  |  |  |
| Range of follow-up (months) | 16 - 39 | 18 - 37 |  |  |  |
| Length of follow-up (months) | 27.15 ± 5.32 | 26.69 ± 6.06 | 0.298 | 0.766 |  |

Values of lengths of follow-up are presented as the means ± standard deviations (SD). No differences were found between the stable groups and the corresponding progressive groups as assessed by Mann‐Whitney tests or two-sample t-tests.

Table S2

**Comparison of the converting rates of Alzheimer's disease at follow-up** **in the aMCI population and rLOD population**

|  |  | Total |  | progressive individuals | | stable individuals |
| --- | --- | --- | --- | --- | --- | --- |
|  |  |  |  |  |  |  |
| aMCI population |  | 33 |  | 6(18.18%) | 27(81.82%) | |
| rLOD  population |  | 39 |  | 10(25.64%) | 29(74.36%) | |
| *χ^2^* |  |  |  | 0.575 | | |
| *p* |  |  |  | 0.448 | | |

Abbreviations, amnesic mild cognitive impairment ; rLOD, remitted late-onset depression.

References

Chen, G., Shu, H., Chen, G., Ward, B. D., Antuono, P. G., Zhang, Z., . . . and Alzheimer's Disease Neuroimaging, I. (2016). Staging Alzheimer's Disease Risk by Sequencing Brain Function and Structure, Cerebrospinal Fluid, and Cognition Biomarkers. *J Alzheimers Dis*. doi: 10.3233/JAD-160537

Chen, J., Duan, X., Shu, H., Wang, Z., Long, Z., Liu, D., . . . Zhang, Z. (2016). Differential contributions of subregions of medial temporal lobe to memory system in amnestic mild cognitive impairment: insights from fMRI study. *Sci Rep, 6*, 26148. doi: 10.1038/srep26148

Chen, J., Shu, H., Wang, Z., Liu, D., Shi, Y., Xu, L., & Zhang, Z. (2016). Protective effect of APOE epsilon 2 on intrinsic functional connectivity of the entorhinal cortex is associated with better episodic memory in elderly individuals with risk factors for Alzheimer's disease. *Oncotarget*. doi: 10.18632/oncotarget.11289

Petersen, R. C., Smith, G. E., Waring, S. C., Ivnik, R. J., Tangalos, E. G., & Kokmen, E. (1999). Mild cognitive impairment: clinical characterization and outcome. *Arch Neurol, 56*(3), 303-308.

Shu, H., Shi, Y., Chen, G., Wang, Z., Liu, D., Yue, C., . . . Zhang, Z. (2016). Opposite Neural Trajectories of Apolipoprotein E 4 and 2 Alleles with Aging Associated with Different Risks of Alzheimer's Disease. *Cereb Cortex, 26*(4), 1421-1429. doi: 10.1093/cercor/bhu237

Tzourio-Mazoyer, N., Landeau, B., Papathanassiou, D., Crivello, F., Etard, O., Delcroix, N., . . . Joliot, M. (2002). Automated anatomical labeling of activations in SPM using a macroscopic anatomical parcellation of the MNI MRI single-subject brain. *Neuroimage, 15*(1), 273-289. doi: 10.1006/nimg.2001.0978

Winblad, B., Palmer, K., Kivipelto, M., Jelic, V., Fratiglioni, L., Wahlund, L. O., . . . Petersen, R. C. (2004). Mild cognitive impairment--beyond controversies, towards a consensus: report of the International Working Group on Mild Cognitive Impairment. *J Intern Med, 256*(3), 240-246. doi: 10.1111/j.1365-2796.2004.01380.x

Xie, W., Song, C., Young, N. L., Sperling, A. S., Xu, F., Sridharan, R., . . . Grunstein, M. (2009). Histone h3 lysine 56 acetylation is linked to the core transcriptional network in human embryonic stem cells. [Research Support, N.I.H., Extramural

Research Support, Non-U.S. Gov't]. *Mol Cell, 33*(4), 417-427. doi: 10.1016/j.molcel.2009.02.004

Young, A. L., Oxtoby, N. P., Daga, P., Cash, D. M., Fox, N. C., Ourselin, S., . . . Alzheimer's Disease Neuroimaging, I. (2014). A data-driven model of biomarker changes in sporadic Alzheimer's disease. *Brain, 137*(Pt 9), 2564-2577. doi: 10.1093/brain/awu176

Chen, J., Chen, G., Shu, H., Chen, G., Ward, B. D., Wang, Z., ... & Initiative, A. S. D. N. (2019). Predicting progression from mild cognitive impairment to Alzheimer’s disease on an individual subject basis by applying the CARE index across different independent cohorts. *Aging (Albany NY)*, *11*(8), 2185.doi: 10.18632/aging.101883.
